# Efficacy, safety, and tolerability of lacosamide in patients with gain-of-function Na_v_1.7 mutation-related small fiber neuropathy: study protocol of a randomized controlled trial–the LENSS study

**DOI:** 10.1186/s13063-016-1430-1

**Published:** 2016-06-30

**Authors:** Bianca T. A. de Greef, Ingemar S. J. Merkies, Margot Geerts, Catharina G. Faber, Janneke G. J. Hoeijmakers

**Affiliations:** Department of Neurology, School of Mental Health and Neuroscience, Maastricht University Medical Center, P. Debijelaan 25, P.O. Box 5800, 6202 AZ Maastricht, The Netherlands; Department of Neurology, St. Elisabeth Hospital, 193 J.H.J. Hamelbergweg, Willemstad, Curaçao

**Keywords:** Small fiber neuropathy, Painful neuropathy, SCN9A gene, Na_v_1.7, Lacosamide, Randomized controlled trial

## Abstract

**Background:**

Small fiber neuropathy generally leads to considerable pain and autonomic symptoms. Gain-of-function mutations in the SCN9A- gene, which codes for the Na_v_1.7 voltage-gated sodium channel, have been reported in small fiber neuropathy, suggesting an underlying genetic basis in a subset of patients. Currently available sodium channel blockers lack selectivity, leading to cardiac and central nervous system side effects. Lacosamide is an anticonvulsant, which blocks Na_v_1.3, Na_v_1.7, and Na_v_1.8, and stabilizes channels in the slow-inactivation state. Since multiple Na_v_1.7 mutations in small fiber neuropathy showed impaired slow-inactivation, lacosamide might be effective.

**Methods/design:**

The Lacosamide-Efficacy-‘N’-Safety in Small fiber neuropathy (LENSS) study is a randomized, double-blind, placebo-controlled, crossover trial in patients with *SCN9A-*associated small fiber neuropathy, with the primary objective to evaluate the efficacy of lacosamide versus placebo. Eligible patients (the aim is to recruit 25) fulfilling the inclusion and exclusion criteria will be randomized to receive lacosamide (200 mg b.i.d.) or placebo during the first double-blinded treatment period (8 weeks), which is preceded by a titration period (3 weeks). The first treatment period will be followed by a tapering period (2 weeks). After a 2-week washout period, patients will crossover to the alternate arm for the second period consisting of an equal titration phase, treatment period, and tapering period. The primary efficacy endpoint will be the proportion of patients demonstrating a 1-point average pain score reduction compared to baseline using the Pain Intensity Numerical Rating Scale. We assume a response rate of approximately 60 % based on the criteria composed by the Initiative on Methods, Measurement, and Pain Assessment in Clinical Trials (IMMPACT) group for measurement of pain. Patients withdrawing from the study will be considered non- responders. Secondary outcomes will include changes in maximum pain score, the Small Fiber Neuropathy Symptoms Inventory Questionnaire, sleep quality and the quality of life assessment, patients’ global impressions of change, and safety and tolerability measurements. Sensitivity analyses will include assessing the proportion of patients having ≥ 2 points average pain improvement compared to the baseline Pain Intensity Numerical Rating Scale scores.

**Discussion:**

This is the first study that will be evaluating the efficacy, safety, and tolerability of lacosamide versus placebo in patients with *SCN9A-*associated small fiber neuropathy. The findings may increase the knowledge on lacosamide as a potential treatment option in patients with painful neuropathies, considering the central role of Na_v_1.7 in pain.

**Trial registration:**

ClinicalTrials.gov, NCT01911975. Registered on 13 July 2013.

**Electronic supplementary material:**

The online version of this article (doi:10.1186/s13063-016-1430-1) contains supplementary material, which is available to authorized users.

## Background

Neuropathic pain is described as “pain caused by a lesion or disease of the somatosensory system” [1]. The prevalence of neuropathic pain in the general population is approximately 7 to 10 % [2, 3]. Neuropathic pain is one of the main symptoms of small fiber neuropathy (SFN), a condition that affects the thinly myelinated Aδ-fibers and the unmyelinated C-fibers. Pain in SFN is mostly described as an itching, burning sensation, usually occurring in a length-dependent pattern, starting in the feet and hands [4]. Body pain is a major contributor to the reduction of quality of life in patients with SFN [5]. In addition to the neuropathic pain, patients with SFN may suffer from autonomic symptoms [6, 7].

Voltage-gated sodium channels have been described to play an important role in neuropathic pain [8]. In approximately 15 % of patients with SFN, gain-of-function mutations in the SCN9A-, SCN10A-, and SCN11A- gene have been reported [9–12]. The SCN9A- gene codes for the voltage-gated sodium channel Na_v_1.7, which is predominantly expressed in the small nociceptive and autonomic neurons. In addition to being associated with SFN, gain-of-function mutations in the SCN9A- gene have been described in the following human pain disorders: inherited erythromelalgia (IEM) and paroxysmal extreme pain disorder (PEPD) [13, 14]. Gain-of-function mutations of the SCN9A- gene were found in 28 % of patients with SFN proven by skin biopsy [9]. In a larger cohort (n = 393), the prevalence of SCN9A- gene mutations in patients diagnosed with SFN based on an abnormal skin biopsy and/or abnormal temperature threshold testing was approximately 9 % [12]. Therefore, Na_V_1.7 appears to be an appropriate target for treatment of different human neuropathic pain conditions, including the *SCN9A*-associated SFN.

Current treatments for pain in patients with SFN are far from satisfactory [15]. Less than 50 % of patients achieve a pain reduction of 50 % [16, 17]. This is possibly due to drugs acting on target sites for which no strong evidence of pathogenicity exists. In addition, commercially available sodium channel blockers are not selective for Na_V_1.7, thereby frequently causing intolerable side effects involving the heart and central nervous system.

Lacosamide is a functionalized amino acid molecule that selectively enhances the slow inactivation of voltage-gated sodium channels and interacts with the collapsin-response mediator protein-2 [18]. Lacosamide differs from other sodium channel blockers because of its unique mechanism of action. It inhibits the currents of hyperexcitable neurons of the voltage-gated sodium channels Na_v_1.3, Na_v_1.7, and Na_v_1.8 by targeting the slow-inactivation state and sparing channels with normal activity [19, 20]. In patients with *SCN9A-*associated SFN, multiple mutations in the SCN9A- gene have shown an impaired slow-inactivation [9], which might potentially be considered a target for the mechanism of action of lacosamide. Therefore, a positive effect on pain reduction in these patients might be expected.

To date, no studies with lacosamide have been performed in patients with SFN. Some evidence exists of lacosamide reducing neuropathic pain and being well tolerated in patients with a painful diabetic neuropathy [21, 22]. The same results were seen in one study with patients with fibromyalgia [23]. However, no robust underlying mechanism has been presented in these studies.

## Methods/design

### Objective

The primary objective of this study is to determine the efficacy and safety of lacosamide versus placebo in patients with *SCN9A*-associated SFN.

### Study design

**Fig. 1:**
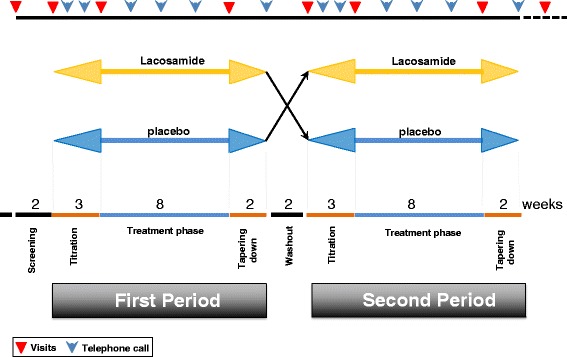
Schematic diagram of study

The Lacosamide-Efficacy-‘N’-Safety in SFN (LENSS) study is a randomized, placebo-controlled, double-blind, crossover-design study (Fig. 1). The study consists of 2 periods. Before the first period, the screening and baseline measurements takes place. Subjects ulfilling the inclusion and exclusion criteria are subsequently randomized to receive lacosamide or placebo. The first period starts with the first titration period of 3 weeks, in which study the medication (lacosamide or equivalent placebo) will be gradually increased. Subsequently, patients enter the first treatment period of 8 weeks. This period is followed by a 2-week tapering period. After a 2-week washout period, the second period, which is executed in the same manner as the first period, begins. Subjects cross over to the alternate arm and undergo the second 3-week titration period, followed by the second treatment period (8 weeks) and a tapering period (2 weeks). In both treatment periods, subjects receive lacosamide 200 mg b.i.d. or placebo. Patients are examined at the study outpatient site at the entry and at the end of both treatment periods (maximum of eight site visits). In addition, subjects are contacted and interviewed in a standardized manner by phone every 2 weeks to determine clinical condition and well-being, and collect data on safety and side effects. A follow-up visit is performed approximately 4 weeks after the last dose of study medication.

The use of drugs such as lamotrigine, carbamazepine, oxcarbazepine, mexiletine, amitriptyline, and topical analgesics (e.g., lidocaine patches, capsaicin patches, and oral/injectable corticosteroids) that act on sodium channels is not allowed during the study period. Participants using these drugs require a washout period of at least five half-lives (90 days for capsaicin patches, which have the longest elimination period of the prohibited medications) prior to the screening visit. Other pain medications can be continued provided the dose and frequency of dosing have been stable over the previous 30 days prior to screening and remains unchanged during the study period. Additional medication is recorded.

#### Study medication

Each treatment phase is preceded by a titration phase. The dose of lacosamide is increased weakly, starting with 50 mg b.i.d. in the first week, followed by 100 mg b.i.d. in the second week, and concluding with 150 mg b.i.d. in the third week. After these 3 weeks, the treatment phase begins for 8 weeks. Based on previous studies and on the dose-related side effects, a dose of 200 mg b.i.d. (or equivalent placebo) has been chosen as the maintenance dose [24]. The subjects start with the maintenance dose (200 mg b.i.d.) at the first day of the treatment phase; therefore, this dose is not included in the titration phase. After the treatment phase, the tapering phase begins, with study medication (lacosamide or placebo) dosage being reduced at once to 100 mg b.i.d. in the first week of the tapering period and then to 50 mg b.i.d. in the second week of the tapering period.

A convenient treatment period of 8 weeks was chosen based on literature findings and assuming that a positive effect of lacosamide should be seen within this period, thereby limiting the burden for patients [23].

### Participants

A total of 25 subjects with genetically proven *SCN9A*-associated SFN are being recruited into the trial. *SCN9A* mutations classified as certainly, probably, or potentially pathogenic are eligible for inclusion [25]. Subjects are recruited at the Maastricht University Medical Center (MUMC+), the Netherlands.

Informed consent is obtained from all participants before any study procedure (e.g., questionnaires or neurological examination) is performed.

#### Inclusion criteria

Subjects must fulfill the following inclusion criteria to be eligible:

1. Male and/or female subjects between 18 and 80 years
2. Presence of a clinical diagnosis of Small Fiber Neuropathy (SFN), with at least two of the following clinical symptoms not otherwise explained:
  Burning feet
  Allodynia
  Diminished pain and/or temperature sensation
  Dry eyes or mouth
  Orthostatic dizziness
  Bowel disturbances (constipation, diarrhea, or gastroparesis)
  Urinary disturbances
  Sweat changes (hyperhidrosis/hypohidrosis)
  Visual accommodation problems and/or blurred vision
  Hot flashes/palpitations
  Impotence or diminished ejaculation or lubrication
3. In addition to the clinical diagnosis of SFN, presence of confirmed abnormality on intra-epidermal nerve fiber density evaluation (IENFD) and/or Quantitative Sensory Testing (QST) [6, 7] *and* a mutation in the SCN9A- gene, confirmed by sequencing. Where possible, in vitro confirmation of the functionality of the mutation should have been performed and documented as has been demonstrated previously [6, 9].
4. Presence of pain due to SFN for at least 3 months prior to screening and an average self-reported pain score of at least 3 during this time.
5. If on analgesic medication, subject must have a stable analgesic medication regimen for a minimum of 30 days before the start of the study and should continue with the same unchanged regimen throughout the study.
6. Evidence of a personally signed and dated informed consent document indicating that the subject (or a legal representative) has been informed of all pertinent aspects of the study.
7. Subjects who are willing and able to comply with scheduled visits, treatment plan, laboratory tests, and other study procedures.

#### Exclusion criteria

Subjects presenting with any of the following cannot be included in the study:

1. Subjects with predominantly signs of large nerve fiber involvement (muscle weakness, loss of vibration sense, or hyporeflexia/areflexia), or clinically significant abnormal nerve conduction studies (NCS)
2. History or presence of illnesses known to cause SFN (excluding diabetes mellitus), including liver, kidney, or thyroid dysfunction, monoclonal gammopathy, connective tissue disorders, sarcoidosis, Sjögren syndrome, amyloidosis, Fabry disease, celiac disease, HIV, and neurotoxic drugs (e.g., chemotherapy)
3. Subjects with other severe pain conditions, which may impair the self-assessment of pain due to SFN
4. Any condition possibly affecting drug intake and absorption (e.g., difficulty in swallowing, gastrectomy and/or bowel resection)
5. History of known alcohol, analgesic, or illicit drug abuse within 12 months of screening
6. Subjects taking medications with potential effect on sodium channel functions, including lamotrigine, carbamazepine, oxcarbazepine, mexiletine, amitriptyline, and topical analgesics (e.g., lidocaine patches, capsaicin patches, and oral/injectable corticosteroids). These medications are prohibited until the end of the study period and require a washout period of at least five half-lives (90 days for capsaicin patches, which is the longest elimination period of the prohibited medications) prior to the screening visit
7. Twelve-lead ECG demonstrating QTcF (Fridericia’s correction) > 450 or a QRS interval > 120 msec at screening. If the QTcF exceeds 450 msec or the QRS exceeds 120 msec, the ECG should be repeated two more times and the average of the three QTcF values should be used to determine the subject’s eligibility.
8. Severe renal impairment (creatinine clearance ≤ 30 mL/min).
9. Treatment with an investigational drug within 30 days (or as determined by the local requirement, whichever is longer) or five half-lives preceding the first dose of study medication.
10. Participation in other studies during the period of current study participation or has surgery planned during the course of the study.
11. Pregnant females, breastfeeding females, or females of childbearing potential not using effective and medically reliable contraception or not agreeing to continue effective contraception for at least 28 days after the last dose of the investigational product.
12. Other clinically significant or unstable, or severe acute or chronic medical, or psychiatric/psychological condition or laboratory abnormality that may increase the risk associated with study participation or investigational product administration or may interfere with the interpretation of study results and, in the judgment of the Investigator, would make the subject inappropriate for entry into this study.
13. In the case of incidental findings, the patient and his/her treating physician will be informed and asked to undertake action if necessary. If a patient does not want to be informed about possible incidental findings and does not want his treating physician to be informed, the patient cannot participate in this study.

#### Randomization

After the screening period, the patients return to our center. To ensure that eligible subjects are compliant, we will verify that at least 5 of the last 7 days of the pain diary are completed. This is important because the pain diary of the screening period is used as the baseline measurement of the primary outcome. Randomization is performed using ALEA data management. This software is provided by the Trans European Network for Clinical Trials Services (http://www.tenalea.com/). The randomization is computer- controlled based on the electronic case report form that is used. A blinded message is send to the investigator and an un-blinded message is send to the pharmacy. Patients are stratified based on the type of the *SCN9A* variant ((1) genetic variant and (2) genetically and functionally confirmed) and on the clinical diagnosis of SFN ((1) abnormal skin biopsy, (2) abnormal temperature threshold testing, and (3) abnormal skin biopsy and abnormal temperature threshold).

#### Blinding

The study is subject-blinded and investigator-blinded until the end of the study. Blinding codes are only broken in emergency situations for reasons of subject safety.

The lacosamide and the matching placebo are provided as 50 mg tablets for oral administration. Tablets are provided in containers. Rescue medication (acetaminophen) is provided in its approved marketed product dress. All medication dispensed to the subjects are to be returned to the investigator and double-checked by the monitor to assure study compliance.

#### Compliance

The following compliance calculation will be applied:

$$ \%\mathrm{Compliance}=\mathrm{number}\ \mathrm{o}\mathrm{f}\ \mathrm{t}\mathrm{ablets}\ \mathrm{t}\mathrm{aken}/\mathrm{number}\ \mathrm{o}\mathrm{f}\ \mathrm{t}\mathrm{ablets}\ \mathrm{expected}\ \mathrm{t}\mathrm{o}\ \mathrm{have}\ \mathrm{t}\mathrm{aken}\times 100. $$

Subjects are coded as being a noncompliant if the percentage compliance according to the above formula is less than 80 % or greater than 120 % study drug compliance.

### Efficacy measurements

The daily Pain Intensity Numerical Rating Scale (PI-NRS) consists of an 11-point numerical scale ranging from 0 to 10, where 0 represents no pain and 10 the worst pain possible. The subjects are asked to complete the PI-NRS twice daily, in the morning and evening, preferably at fixed time points. In addition, the daily sleep interference scale (DSIS) is completed every day on awakening in the morning. The DSIS consists of an 11-point numerical scale ranging from 0 (pain does not interfere with sleep) to 10 (pain completely interferes with sleep) and is used to determine sleep quality. At each assessment, the following additional questionnaires are completed: the Neuropathic Pain Scale (NPS), Small Fiber Neuropathy Symptom Inventory Questionnaire (SFN-SIQ), Patient’s Global Impression of Change (PGIC), and the generic Short Form (36) Health Survey (SF-36).

### Outcome measurements

The chosen outcomes are largely based on the international criteria advised by the IMMPACT group for measurement of pain [26].

#### Primary outcome

The primary efficacy endpoint is defined as the proportion of patients demonstrating a 1-point average pain score reduction compared to baseline using the PI-NRS. A 1-point change on the PI-NRS is considered the minimum clinically important difference (MCID) according to the unified rule of ½ x standard deviation (SD) and recommendations given by the IMMPACT group [26, 27].

#### Secondary outcome

Secondary outcomes include changes seen in the maximum pain score on the PI-NRS, the NPS, DSIS, PGIC, SFN-SIQ, SF-36, adverse events, laboratory safety tests (e.g., hematology and clinical chemistry), blood pressure (BP), pulse rate (PR), and electrocardiogram (ECG).

Sensitivity analyses include assessing the proportion of patients having ≥ 2 points average pain improvement compared to their baseline PI-NRS scores.

#### Data management

An electronic case report file (eCRF) is used for each patient to collect all data. To host the eCRF, MACRO electronic data capture is used, powered by InferMed Ltd, London, UK. It has been designed to support compliance with the requirements of relevant regulatory bodies including International Council for Harmonisation (ICH) Good Clinical Practice (www.infermed.com). Assessments start at the screening visit. In addition, assessments at site visits and by phone are performed according to the scheme presented in Fig. 1 and include a standardized interview to determine the patient’s clinical condition and well-being, assess the various questionnaires, determine compliance, assess laboratory results, and at predefined moments, perform ECG. During each contact (visit or by phone), adverse events, and concomitant medication are discussed. At each visit, the diary and the remaining medications are collected. A summary of the assessments made during visits and telephone calls is provided in Table 1.

**Table 1:** Schedule of assessments throughout the study

Protocol Activity	Screen (Week -4-0)	Base-line/ Randomization	Titration (Week 1-3)		Treatment Period 1 (Week 4- 11)		Tapering Week 12-13	Washout Week 14-15	Titration (Week 16-18)		Treatment Period 2 (Week 19- 26)		Tapering Week 27-28	Follow-up Week 33
Clinic Visit^a^	V1	V2	T1-2	V3	T3-T5	V4	T6	V5	T7-T8	V6	T9-T11	V7	T12	V8
Informed Consent	X													
Inclusion/Exclusion criteria	X													
Randomization^b^		X												
Medical History	X													
Demography	X													
Physical Examination (Full)	X													
Physical Examination (Brief)		X		X		X		X		X		X		
Weight and Height	X													
Safety Laboratory Tests^c^	X			X		X		X		X		X		
FSH^d^	X													
HbA1c blood test^e^	X													
12-lead ECG	X			X		X		X		X		X		
BP (supine and standing) and PR	X			X		X		X		X		X		
Daily Pain Diary (PI-NRS)^f^		X												X
Daily Sleep Interference Scale (DSIS)^g^		X												X
Neuropathic Pain Scale (NPS)	X	X	X	X	X	X	X	X	X	X	X	X	X	
SFN-SIQ Questionnaire	X	X	X	X	X	X	X	X	X	X	X	X	X	
Patient Global Impression of Change		X	X	X	X	X	X	X	X	X	X	X	X	
SF-36		X				X		X				X		
Adverse Event Monitoring^h^		X												X
Concomitant Medication^h^		X												X
Dispense Study Medication^i^		X		X		X		X		X		X		
Retrieve Study Medication dispensed at previous study visit				X		X		X		X		X		X
Dispense Rescue Medication		X		X		X		X		X		X		
Retrieve Rescue Medication dispensed at previous study visit				X		X		X		X		X		X
Dispense and instruct on Daily Pain Diaries	X													
Retrieve Daily Pain Diary	X	X		X		X		X		X		X		X
Study medication compliance check				X		X		X		X		X		X

BP; blood pressure, ECG; electrocardiogram, FSH; follicle-stimulating hormone, HbA1c; hemoglobin A1c, NPS; neuropathic pain scale, PI-NRS; pain intensity numerical rating scale, PR; pulse rate, SFN-SIQ; small fiber neuropathy symptom inventory questionnaire, T; telephone call, V; visit, SF-36; short form 36

^a.^ Visit/study activity window can be ± 2 days

^b.^ Subjects will be randomized provided they fulfill study selection criteria

^c.^ Safety laboratory tests include hematology and clinical chemistry

^d.^ Females who are 45-60 years of age who are amenorrheic for at least 1 year

^e.^ HbA1c test to be performed in subjects with diabetes and at investigator’s discretion for subjects who do not have a clinical diagnosis of diabetes but present with hyperglycemia on safety lab tests

^f.^ Daily Pain Diary (PI-NRS) to be completed by subject twice daily (morning and evening pain scores) and reviewed by study personnel at scheduled clinic visits

^g.^ Daily Sleep Interference Scale (DSIS) to be completed by subject once daily on waking starting morning after Screening Visit (V1)

^h.^ Adverse events and concomitant medication will be monitored during the entire study

^I.^ Full dosing instructions will be provided and the first dose of study medication will be taken on the evening of the SECOND study visit (V2)

Privacy of the patients is guaranteed; stored data and materials are only identifiable to the person by a sequentially assigned subject number. The handling of personal data complies with the Dutch Personal Data Protection Act (De Wet Bescherming Persoonsgegevens, WBP). The SPIRIT checklist and figure for this study protocol are shown in Additional files 1 and 2.

#### Safety reporting

Adverse events are recorded and monitored. The principal investigator is to be informed immediately in case of any serious adverse event (SAE). Every SAE is reported to the Ethics Committee. Suspected unexpected serious adverse reactions (SUSAR) are also reported. Furthermore, all SUSARs are expedited to the competent authorities in other Member States, according to the requirements of the Member States.

### Statistical analysis

#### Sample size

A 1-point change on the average PI-NRS compared to baseline is considered as the MCID [26, 27]. We assume a response rate of approximately 20 % in the placebo-treated group, based on a meta-analysis of the placebo effect in pain studies in which the effect varied from 7-37 %, with a 50 % pain reduction in 16 %. In the lacosamide-treated group, we assume a response rate of approximately 60 % based on the IMMPACT criteria in which the clinical relevant pain reduction may be less than 50 %. Fixing a two-sided alpha at 5 %, a sample size of 22 patients is required per treatment group to show efficacy with 80 % power between the two groups (chi-square test). Assuming a dropout rate of approximately 10 % (two to three patients), a total of 25 subjects will be needed per treatment group (in a parallel study). Since the number of patients with *SCN9A*-associated SFN is limited, a crossover design was chosen to fulfill sample size requirements with the inclusion of 25 subjects.

#### Type of analysis

The analyses are performed on the intention-to-treat (ITT) population, defined as all patients who received at least one dose of randomized study medication and had at least one post-baseline assessment. Patients withdrawing from the study are considered as nonresponders. The comparison of the proportion of patients in both groups (lacosamide versus placebo) reaching the predefined cut-off is estimated using Kaplan-Meijer serial time series graphs with a log-rank test.

For secondary efficacy endpoints, the treatment differences for change from baseline in selected outcome measures, according to the predefined inquiries, are analyzed using nonparametric tests. The statistical tests used depend on the type of data. Analyses of safety parameters are performed on the safety set (SS), which includes all randomized patients who took at least one dose of trial medication. Individual missing data are assigned using a last observation carried forward approach. Other missing-data treatment methods are performed (for example single and multiple imputation) to test which of these methods is the best according to sensitivity analyses.

### Discussion

In this study, the efficacy, safety, and tolerability of lacosamide in patients with pain due to *SCN9A*-associated SFN are studied. Despite earlier studies performed with lacosamide in painful conditions, no study to date has been done in patients with *SCN9A*-associated SFN. This is interesting because most of them harbor an electrophysiological mechanism of pain induction through impaired slow-inactivation of the voltage-gated sodium channel Na_V_1.7 [28], which might be a potential target for lacosamide, taking into account its unique mechanism of targeting the slow-inactivation [19]. Therefore, a positive effect on pain reduction in these patients might be expected.

With lacosamide, we hope to find a new treatment option for the excruciating pain often reported by patients with SFN. This is the first pilot study that aims to show the efficacy, tolerability, and safety of lacosamide in this specific cohort of patients with *SCN9A*-associated SFN.

If lacosamide proves to be effective in SFN patients with a Na_v_1.7 mutation, this might be a viable option for patients with painful neuropathies or with neuropathic pain in general, considering the central role of Na_v_1.7 in pain. The first results of the study are expected in mid-2017.

### Trial status

Participant recruitment for this trial is ongoing. Recruitment began in November 2014 and is expected to end mid-2016.

## Abbreviations

b.i.d., bis in die (twice daily); BP, blood pressure; DRG, dorsal root ganglion; DSIS, Daily Sleep Interference Scale; ECG, electrocardiogram; eCRF, electronic case report file; FSH, follicle-stimulating hormone; ICH, International Council for Harmonisation; IEM, inherited erythromelalgia; IENFD, intraepidermal fiber density; IMMPACT, Initiative on Methods, Measurement, and Pain Assessment in Clinical Trials; ITT, intention-to-treat; LENSS study, The Lacosamide-Efficacy-‘N’-Safety in Small fiber neuropathy study; MCID, minimum clinically important difference; MUMC, Maastricht University Medical Center; Na_v_1.7, voltage-gated sodium channel 1.7; NCS, nerve conduction studies; NPS, neuropathic pain scale; PEPD, paroxysmal pain disorder; PGIC, patient global impression change; PI-NRS, Pain Intensity Numerical Rating Scale; PR, pulse rate; QST, quantitative sensory testing; SAE, serious adverse event; *SCN9A*-associated SFN, gain-of-function SCN9A mutation-related small fiber neuropathy; SD, standard deviation; SF-36, Short Form (36) Health Survey; SFN, small fiber neuropathy; SFN-SIQ, Small Fiber Neuropathy Symptom Inventory Questionnaire; SS, safety set; WBP, Wet Bescherming Persoonsgegevens

## Additional files

Additional file 1: Checklist SPIRIT. (DOC 121 kb) Additional file 2: Figure of content for the schedule of enrolment, interventions, and assessments. (DOCX 95 kb)
