# Supplementary material for: Efficacy, safety, and tolerability of lacosamide in patients with gain-of-function Nav1.7 mutation-related small fiber neuropathy: study protocol of a randomized controlled trial–the LENSS study
Source: Trials. 2016 Jun 30;17:306. doi: 10.1186/s13063-016-1430-1 (PMC4929773; doi:10.1186/s13063-016-1430-1)
Supplement: Additional file 2: — Figure of content for the schedule of enrolment, interventions, and assessments. (DOCX 95 kb) [file 13063_2016_1430_MOESM2_ESM.docx]

**Additional file 2. Figure of content for the schedule of enrolment, interventions, and assessments.***

|  | **STUDY PERIOD** | | | | | | | | | | | | | |
| --- | --- | --- | --- | --- | --- | --- | --- | --- | --- | --- | --- | --- | --- | --- |
|  | **Enrolment** | **Allocation** | **Post-allocation** | | | | | | | | | | | **Close-out** |
| **TIMEPOINT**** | ***-v_1_*** | **0*(v_2_)*** | ***t_1-2_*** | ***v_3_*** | ***t_3-5_*** | ***v_4_*** | ***t_6_*** | ***v_5_*** | ***t_7-8_*** | ***v_6_*** | ***t_9-11_*** | ***v_7_*** | ***t_12_*** | ***v_8_*** |
| **ENROLMENT:** |  |  |  |  |  |  |  |  |  |  |  |  |  |  |
| **Eligibility screen** | X |  |  |  |  |  |  |  |  |  |  |  |  |  |
| **Informed consent** | X |  |  |  |  |  |  |  |  |  |  |  |  |  |
| **Medical history** | X |  |  |  |  |  |  |  |  |  |  |  |  |  |
| **Demography** | X |  |  |  |  |  |  |  |  |  |  |  |  |  |
| **Allocation** |  | X |  |  |  |  |  |  |  |  |  |  |  |  |
| **Randomization** |  | X |  |  |  |  |  |  |  |  |  |  |  |  |
| **INTERVENTIONS:** |  |  |  |  |  |  |  |  |  |  |  |  |  |  |
| ***Lacosamide*** |  |  |  |  |  |  |  |  |  |  |  |  |  |  |
| ***Placebo*** |  |  |  |  |  |  |  |  |  |  |  |  |  |  |
| **ASSESSMENTS:** |  |  |  |  |  |  |  |  |  |  |  |  |  |  |
| **Physical examination** | X | X |  | X |  | X |  | X |  | X |  | X |  |  |
| **Safety Laboratory tests** | X |  |  | X |  | X |  | X |  | X |  | X |  |  |
| **12-lead ECG** | X |  |  | X |  | X |  | X |  | X |  | X |  |  |
| ***Daily Pain Diary (PI-NRS)*** | X |  |  |  |  |  |  |  |  |  |  |  |  | X |
| ***Daily Sleep Interference scale (DSIS)*** | X |  |  |  |  |  |  |  |  |  |  |  |  | X |
| ***Neuropathic Pain Scale (NPS)*** | X | X | X | X | X | X | X | X | X | X | X | X | X |  |
| ***SFN-SIQ Questionnare*** | X | X | X | X | X | X | X | X | X | X | X | X | X |  |
| ***Patient Global Impressions of Change (PGIC)*** |  | X | X | X | X | X | X | X | X | X | X | X | X |  |
| ***SF-36*** |  | X |  |  |  | X |  | X |  |  |  | X |  |  |
| ***Adverse Event Monitoring*** | X |  |  |  |  |  |  |  |  |  |  |  |  | X |
| ***Concomitant Medication*** | X |  |  |  |  |  |  |  |  |  |  |  |  | X |

*Recommended content can be displayed using various schematic formats. See SPIRIT 2013 Explanation and Elaboration for examples from protocols.

**List specific timepoints in this row.
